# Developing a Tool to Support Communication of Parental Concerns When a Child is in Hospital

**DOI:** 10.3390/healthcare4010009

**Published:** 2016-01-13

**Authors:** Gemma Heath, Hermione Montgomery, Caron Eyre, Carole Cummins, Helen Pattison, Rachel Shaw

**Affiliations:** 1 Department of Psychology, School of Life and Health Sciences, Aston University, Birmingham B4 7ET, UK; h.m.pattison@aston.ac.uk (H.P.); r.l.shaw@aston.ac.uk (R.S.); 2 Birmingham Children’s Hospital, Steelhouse Lane, Birmingham B4 6NH, UK; hermione.montgomery@bch.nhs.uk (H.M.); caron.eyre@bch.nhs.uk (C.E.); 3 Institute of Applied Health Research, University of Birmingham, Birmingham B15 2TT, UK; c.l.cummins@bham.ac.uk

**Keywords:** paediatrics, communication, quality improvement, patient safety, parents

## Abstract

The involvement of parents in their child’s hospital care has been strongly advocated in paediatric healthcare policy and practice. However, incorporating parental worries about their child’s condition into clinical care can be difficult for both parents and healthcare professionals. Through our “Listening To You” quality improvement project we developed and piloted an innovative approach to listening, incorporating and responding to parental concerns regarding their child’s condition when in hospital. Here we describe the phases of work undertaken to develop our “Listening To You” communications bundle, including a survey, literature review and consultation with parents and staff, before findings from the project evaluation are presented and discussed.

## 1. Introduction

When a child’s health unexpectedly deteriorates, parents often express that they had “known” or “felt” that their child’s symptoms were more serious than the health professionals had understood [1]. Staff also recognise barriers to effective communication with parents regarding their concerns [2]. Active elicitation and reaction to such parental insight could facilitate the early identification of clinical deterioration [3]. Systems such as the Paediatric Early Warning Score (PEWS) are used by professionals to recognise and respond to patients’ symptoms [4] but currently there is no systematic way in which parents can contribute to this assessment. New systems enabling families to initiate a rapid medical response for deteriorating children are also starting to emerge [1,3,5], however, evidence for the impact and effectiveness of these family-led systems has yet to be fully assessed [6,7]. Our quality improvement project aimed to develop a tool to support parents in communicating and escalating concerns about their child’s clinical condition when in hospital. Understanding parental concern as an indicator of clinical deterioration and empowering parents to speak-up when they are worried has recently been underscored within the context of improving care quality and safety, particularly in terms of preventing avoidable harm in children [8].

## 2. Background

Parental involvement in children’s hospital care has been advocated in the UK since the Platt report in 1959 [9]. Current policy recommends parents are treated as active partners in their child’s care [10] and that no decision is made about a child’s care without the input of parent and child [11]. This Family-Centred Care approach has long been the ethos in child health settings [12]. Valuing parents’ knowledge and experiences of their child through working in partnership with families is a fundamental feature of Family-Centred Care, but it could also be a valuable asset in monitoring the child’s clinical condition [13]. However, studies demonstrate challenges in supporting and incorporating parental involvement in paediatric healthcare [14,15,16]. Lack of communication, entrenched professional practices, limited role negotiation and ambiguous care boundaries mean that “partnership in care” can be difficult to achieve in practice [15,17,18].

Paediatric inpatients often display physiological or behavioural signs of clinical deterioration prior to becoming critically ill [19]. Reliable early identification of such symptoms can help to prevent life threatening events [20,21]. Consequently, the Confidential Enquiry into Maternal and Child Health report “Why Children Die” recommended a “standardised and rational monitoring system with imbedded early identification systems for children developing critical illness—an Early Warning Score” [22]. Using structured observation and recording, Paediatric Early Warning Scores (PEWS) enable health professionals to assess and quantify changes in a patient’s condition, triggering patient review above a pre-defined threshold [4,23]. Hospitals that have implemented PEWS with a rapid response team have demonstrated improved patient outcomes such as reduction of cardiac arrest and mortality [24,25].

Studies also suggest that clinical intuition or “gut feeling” plays an important part in the health professional’s recognition of serious illness and patient deterioration [26,27,28,29]. Such intuitive knowing is described as a rapid, subtle and contextual process which integrates and makes sense of, multiple complex pieces of information [29,30]. Key to this is “knowing the patient” [31,32]. While health professionals may be able to build up a relationship with the patient over time so that subtle changes in their appearance, behaviour or condition can be recognised, patients’ relatives are likely to be the experts in “knowing the patient” and may therefore be well placed to raise concerns about changes in their condition before abnormal vital signs become apparent [26,27]. In recognition of this, hospitals are starting to explore the role of families in detecting, alerting and activating medical review of the deteriorating patient [1,2,3,5,33]. Findings from a recent review of the impact of family-activated rapid response teams/systems [7] indicated earlier intervention for patient deterioration, leading to improved health outcomes. Components of effective systems included clear information on how to report family concern (e.g., calling a dedicated telephone number based on a specified criteria to request a rapid response team), that was delivered in multiple ways (e.g., poster, leaflets, and videos) and accompanied by explanation and support from health professionals. Implementation of family-activated response systems has also been found to strengthen partnerships between families and professionals, thereby facilitating Family-Centred Care [7]. Despite potentially promising results, evidence for this kind of approach in paediatrics remains limited [6].

At the time of the study, there were few guidelines on purposefully involving parents in hospital care processes [15,34] and no published frameworks for eliciting, incorporating or responding to parental concern regarding their child’s clinical condition. This study aimed to fill this gap by developing a tool to support communication and escalation of parental concern when a child is in hospital.

## 3. Local Problem

While understanding the importance and value of working in partnership with parents to identify and understand the subtle changes in a child’s health condition, health professionals at a UK Children’s Hospital recognised that different parents, families and carers voiced their observations and concerns in different ways and at different times. Variation in the way that parents/carers raised their concerns, in conjunction with the lack of a unified approach in the way staff listened or responded to those concerns, meant that some families failed to have their concerns acknowledged with the seriousness that was required. This inconsistency was resulting in poor patient experience and in some cases, a failure to escalate care where needed. Recognising the problem, staff wanted to find a way of actively supporting parents to communicate concerns regarding their child’s clinical condition (particularly signs of deterioration), in a way that was appropriate for them and in a way that could be acknowledged, documented and escalated. It became clear that there was a need to formalise the hospital’s approach to listening and responding to parents’ observations and/or concerns regarding their child’s clinical deterioration when in hospital. The aim of the “Listening To You” Project was to develop a tool to support communication of parental concerns and appropriate escalation of care, thereby activating earlier intervention and improving care quality and safety.

In this article, we describe the phases of work undertaken to understand the problem and explore potential solutions and then how we used this insight to develop a communications bundle for parents and staff. The intervention was revised in light of service-user feedback before being piloted. Findings of this pilot are presented and discussed. The questions guiding our quality improvement project were:

- How can we support parents to raise concerns about their child’s health in a standardised and unified way?
- How can we ensure concerns are listened to and care is escalated when needed?

## 4. Methods

### 4.1. Setting

Birmingham Children’s Hospital NHS Foundation Trust (BCH) is a large, UK specialist hospital providing secondary and tertiary inpatient and outpatient care to children and young people locally, nationally and internationally. The hospital has 360 beds across 34 specialities, including a 31 bed paediatric intensive care unit (PICU). Per year, it has 257,173 patient visits, with 42,507 inpatient admissions [35]. Parents are invited to stay with their child during their admission and are provided accommodation free of charge either within the hospital or within a purpose built facility located adjacent to the hospital.

### 4.2. Planning the Intervention

#### 4.2.1. Phase 1: Understanding the Problem and Exploring Potential Solutions

##### National Survey of Practice

A survey of current practice at hospitals providing paediatric health services across the UK was conducted to identify existing tools and to examine staff perceptions of their use, function and effectiveness. Sixteen hospitals were identified as accepting paediatric intensive care admissions. Health professionals on the medical and surgical ward at each hospital were contacted by a research assistant by telephone. Of the 16 hospitals contacted, two were excluded due to (i) providing cardiac and thoracic services only; and (ii) having only one ward which dealt with both medical and surgical admissions. A total of 31 wards, across 14 hospitals were contacted. Two wards declined to take part. Survey data were collected over a period of one month (July 2013) via telephone or email. Findings revealed that none of the hospitals had a formal way of eliciting or assessing parental concerns, or any way of parents accessing the medical team directly, other than by seeing them on the ward. The majority of hospital sites had an established Paediatric Early Warning System (or similar form of early warning score) in place, but parents had no access to this. Four of the wards incorporated parental concern into the PEWS score, which added a point if parents were concerned. From this survey, we concluded that there was no framework available within the UK National Health Service (NHS) for quantifying and managing parental concerns to help ensure that parental input is used consistently.

##### Literature Review

A systematic review of published literature was conducted to examine the evidence on supporting communication between health professionals, patients and their families while in hospital. In brief, studies were identified by searching five data-bases (BioMed, PubMed, Web of Knowledge, ScienceDirect, Aston University e-Library). Key search terms were adapted for each data-base, but included variations on: health professional; parent; child; experiences or support or needs; communication; escalation of care. Studies were included if they examined the facilitators or inhibitors of communication between patients, families and healthcare professionals particularly focusing on the expression of concern and escalation of care. To be as inclusive as possible, the search was not restricted to paediatrics or to any particular methodological approach. Identified studies were appraised for quality using a tool developed for assessing studies with diverse designs [36] and synthesised narratively. Following screening, 30 papers were included in the review (see flow diagram in supplementary materials); however, the majority of studies identified were based in adult care. Furthermore, the focus was on communication initiated by health professionals rather than by parents. Table S1 presents the characteristics of included studies. Key findings indicated a need for health professionals to listen to patients and to establish a rapport in order to facilitate dialogue throughout the patient’s stay in hospital [37,38,39]. Patient satisfaction levels were higher when professionals offered emotional as well as physical care, particularly when in hospital long-term [40,41,42,43]. Having a written/visual prompt or time to think about potential conversations was suggested to help patients/parents articulate their concerns to health professionals [38,44,45,46]. Training for staff was recommended to help raise staff levels of self-efficacy in providing holistic care, but increased exposure to productive communication in complex clinical scenarios was also likely to be required [47]. From this review, we concluded that further research was needed to investigate parent-initiated communication regarding observations or concern about their child’s clinical condition in paediatric hospital settings.

##### Consultation with Parents and Health Professionals

Given the lack of existing evidence on our topic, it was important to obtain a more in-depth understanding from families and staff on their experiences of parents raising concerns regarding their child’s deteriorating condition and views on approaches to facilitating this kind of communication. We conducted semi-structured interviews with 10 parents and 14 health professionals (doctors, nurses, family advocates) recruited from wards covering a range of clinical areas (cardiology, hepatology and gastroenterology, medical) (see Table 1 for interview questions). Qualitative data were coded by two researchers and analysed thematically [48]. Themes were discussed with members of the wider project team and refined accordingly.

Findings revealed that parents had a strong desire to be involved in their hospitalised child’s care and given their expert knowledge of their child, were considered well placed to identify signs of clinical deterioration.

“As a mother, its instinct isn’t it ... if your baby is unwell … even if he’s got a temperature you’d know, as a mother, you’d know just looking at them because it’s instinct really” (Parent 1)

However, parents required clarity on their role within the multi-disciplinary team and support in articulating their concerns.

“It’s hard to know who you speak to, that’s the thing. Because there’s been many occasions where we’ve felt that we wanted to do that but you don’t know who to go to” (Parent 2)

“We kept saying ‘Oh, there’s something not quite right’ and we just … well I was like, ‘we don’t know what it is but he’s just not right’.” (Parent 3)

By spending time accompanying their child on the ward, parents established their own steps for managing concerns through trial and error. They learnt who to go to, at what times, and what to say to get their concerns “heard”:

“I tried to use medical language and terms that obviously you pick up over the years … to gain credence and respect from the people that were looking after (daughter)” (Parent 8)

“In the end we felt our only option was to walk onto PICU to request help from the Consultant there, who came up to help us. No matter how many times I told the nurses, they weren’t doing anything, they weren’t listening to me” (Parent 9)

However, it was acknowledged that this approach was highly dependent on the amount of time spent on the ward, relationships developed with staff and confidence of the parents.

Some parents also had reservations about escalating their concerns for fear of compromising relationships with staff and potentially, the care that their child received.

“When you are in that situation, you don’t want to fall out ... you don’t want to fall out with anybody because your child is in their care, you want the best care for your child. So you don’t want to upset them” (Parent 8)

For staff, distinguishing between urgent clinical concern and “normal” parental anxiety, and managing parental expectations were perceived as challenging and required expert skills to be dealt with effectively.

“It’s very difficult to work out are they just stressed because their kid has been admitted to hospital or are they genuinely concerned that their child is clinically deteriorating” (Nurse 5)

**Table 1:** Semi-structured interview guide.

**Interviews with Parents**
During the time your child was in hospital, were you ever concerned about her/his condition getting worse? What sort of information, if any, have you had about how to read the signs that something might be wrong with your child? What is/was your experience of raising concerns with a member of hospital staff? How did you talk to staff about your concerns while your child was in hospital? What advice would you to give to other parents in a similar situation? What would you change to enable parents to talk to staff more effectively about their concerns?
**Interviews with Staff**
What is your experience of parents expressing concern about their child’s condition? What sort of language do parents use to express their concern? How do you manage parents’ concern when you think there is no clinical evidence to cause concern? Is there a formal pathway for parents to raise their concerns on your ward/speciality? How do you feel about escalating concerns on behalf of parents? Do you record parental concerns? If so where/how? What do you think are the biggest challenges in listening to parents’ concerns?

All participants recognised the importance of staff being transparent about their responses to parental concerns and involving parents in any actions or decisions taken.

Parents recommended that professionals actively create opportunities for communication by inviting them to discuss problems or worries. They further suggested that those families who were new to the ward would benefit from being provided with the kinds of information they acquired over time (e.g., how to distinguish professionals, when to raise concerns). Other ideas included parents posting anonymous messages about their worries for practitioner response, and displaying a formalised care escalation pathway on posters or videos. Staff felt that the development of a tool to support parental communication would be helpful, but they were cautious about implementation; highlighting that it was important to think carefully about the content and operationalisation of any tools.

From this consultation activity, we concluded that pathways for escalating parental concerns needed to be clear and consistent, with guidance provided to families on how to communicate worries about their child’s condition to staff, on the information that would assist health professionals in their clinical assessments and on the procedure for parent activated care escalation where required. Findings also indicated that professionals required support in eliciting signs of parental worry and to “actively listen” and respond to parents’ concerns.

#### 4.2.2. Phase 2: Intervention Development

**Figure 1:**
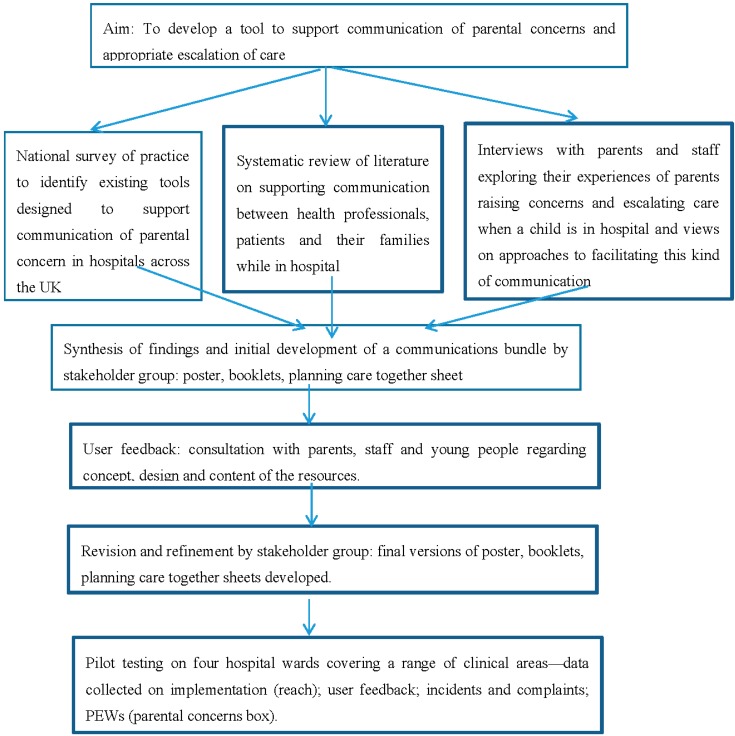
Intervention development and pilot testing.

Findings from data gathered within phase 1 were synthesised by the team, using a matrix to compare findings. Core themes related to the mastery of communication skills, barriers to communication (e.g., lack of time; lack of guidance on defining and managing parental concerns; anxious parents; not feeling listened to) and ideas for potential solutions (e.g., documenting concern; providing a direct phone line or dedicated email service; providing written information and guidance; policy or formal channels for communication of parental concerns/family-initiated escalation of care). These findings were then discussed within a meeting of stakeholders including nurses, psychologists and researchers (See Figure 1). It was agreed that a “communications bundle” for parents and staff would be developed around the concept of “Listening”. Key principles underpinning the tools included: valuing parents’ knowledge of their child; supporting parents to raise concerns; listening and incorporating parental expertise into clinical care; actively eliciting and responding to parents’ concerns; communicating actions taken. Resources comprised:

*Parents:* a poster and booklet for parents, guiding them on how to raise their concerns with staff. These resources, entitled “Listening To You” were written in language that was accessible for parents and included strategies for helping parents to describe what was making them worried (using a diagram and example clinical presentations), a step-by-step guide to escalating concerns to more senior members of staff, and tips on initiating conversations with staff, describing concerns and negotiating solutions or plans. The booklet aimed to give parents “permission” to raise their concerns with staff, clear channels for doing so and a consistent pathway for escalating their concerns if needed.

*Parents and staff:* a “Talking to staff about your worries: Planning Care Together” sheet was also developed to aid parents in sharing their concerns, initiating conversations with staff and for negotiating what they would like staff to do about it. This tool was designed for parents and staff to share, discuss and document parental concerns, that both parties would agree to and sign. It would then be filed with the child’s PEWs chart in the clinical notes and kept as a record of the Planning Care Together conversation.

*Staff:* A booklet for staff outlining their role in the “Listening To You” approach, and presenting strategies for active listening (e.g., summarising, listening for feelings, probing for information) and communicating with parents regarding their concerns (e.g., avoiding medical jargon, feeding back actions). The Situation, Background, Assessment, Recommendation (SBAR) acronym was recommended for helping to elicit information from parents and escalating concerns to doctors. The Illness severity, Patient summary, Action list, Situation awareness and contingency planning, Synthesis by receiver (IPASS) acronym was recommended for escalating the child’s care to doctors or to the Paediatric Assessment Clinical Intervention and Education (PACE) team. In addition, staff were informed on the guidance parents were being given regarding communicating their concerns.

Following development of the “Listening To You” communications bundle, all resources were reviewed by parents (*n* = 7), health professionals (*n* = 5) and adolescent patients (*n* = 3) on hospital wards (medical, liver and small bowel, cardiac); at the hospital-based “Tea@3” parents gathering; and within the hospital Young Persons Advisory Group. Views were collected qualitatively on the concept, content and design of the tools. Feedback was generally positive. Parents expressed that they sought something “*tangible*” that would give them “*confidence and power*” to raise concerns with staff. They confirmed that the prototype resources would be of use to parents and provided suggestions for improvement. Young people contributed to the design of the resources, in particular the name and logo. All agreed that “Listening To You” was an appropriate and helpful name. Staff commented on the resources for both staff and parents. Comments were collated and then discussed within a second stakeholder meeting, where each resource was refined. This led to the development of the final communication bundle for parents and staff (see supplementary materials for resources).

### 4.3. Methods of Evaluation

The “Listening To You” communication bundle was planned to be piloted on four wards covering a range of clinical areas. Clinical members of the project team took responsibility for approaching ward managers on each pilot ward to help launch the project in their areas. Resources were evaluated in terms of implementation and effectiveness from the perspective of intervention users (user feedback); impact on the reporting of serious incidents and complaints; and improvements to parent/patient experience.

Implementation: The number and characteristics of people aware of and using the tool was evaluated on a daily basis using a questionnaire which was verbally administered by members of the project team to parents and staff. The aim was to establish if, where, when and how often the “Listening To You” communication bundle (poster, booklets, Planning Together sheet) was being used.

User feedback: Where the resources had been used, parents and staff were invited to complete questions regarding their experiences of using them and what improvements or modifications could be made. User feedback was also collected informally by members of the project team through conversations with staff and parents on the wards, at meetings and through third parties such as the PICU Family Liaison nurses.

Incidents and complaints: The project team collected information from the Hospital Governance Department relating to the number of Serious Incidents Requiring Investigation (SIRIs), formal complaints and Patient Advice and Liaison Services (PALS) queries regarding parental concerns and the escalation of care/concerns.

Paediatric Early Warning Score Sheets (parental concerns box): Project team members spent time on two wards looking at the completion of their PEWS parental concerns boxes and measuring the number of completed boxes. This was considered to be helpful for understanding how comfortable parents and staff felt about raising and discussing parental concerns.

### 4.4. Analysis

Data were collected by project team members. Quantitative data were summarised descriptively and qualitative data analysed thematically.

## 5. Results

The communication bundle was piloted for one month (December 2014) on four wards: cardiac, oncology, respiratory and long term conditions. Patients on these wards ranged from neonates to adolescents and some had learning disabilities. Length of stay ranged from less than one week to six weeks and over. Posters were prominently displayed within ward areas and family rooms. Booklets and “Planning Together” sheets were given to families by clinical staff. Following a request from nurses, the pilot was extended to parents with children on the Paediatric Intensive Care Unit (PICU). These parents were recognised as having a definite need for the “Listening To You” resources and staff felt they would be of particular use in this setting. However, due to the sensitive nature of PICU, evaluation was largely based on informal conversations with parents and staff using the tools. PICU Family Liaison nurses regularly reported to the project team on the effectiveness of the intervention. Table 2 presents a summary of the results.

**Table 2:** Evaluation results.

**Parents**		**Yes**	**No**
**Have you seen**	Poster	24	27
	Booklet	20	31
**Have you used**	Resources	3	48
**If you’ve not used resources, why?**	Not seen	30	
	Not needed	11	
	Concern resolved verbally	6	
	Other	1	
**If you have used resources, what did you think?**	Were they easy to use?	1	
	Did they help communication with staff?	0	
	Did they give you more confidence to raise your concern with staff?	2	
**Types of concerns raised**	No feedback from doctors		
**Other comments**	Would be good if they had concerns		
	If younger parent or first timer it would be more useful		
	Good idea, especially to write down and then have to hand when the right person comes around		
**Staff**		**Yes**	**No**
**Did you know about the project?**		38	11
**Have any parents raised concerns with you using these resources?**		4	37
**If you have used the staff resources, what did you think?**	Were they easy to use?	23	0
	Did they help communication with parents?	17	0
	Did they give you more confidence to discuss parent’s concerns with them?	22	1
**Do you think parents feel their child is safer knowing they can raise concerns?**		20	0
**Has the tool increased your workload?**		3	27
**Types of concerns raised**	Feeding reassurance		
	Feeding *vs.* weight gain		
	Non-medical		
**Other comments**	Need to work with info for a couple of weeks before can make suggestions		
	Parents will feel empowered because they can write it down		
	Doctors need to talk to parents more		
	Ward plan to add resources to admissions documentation		
	Increased confidence especially for new and newly qualified staff		
	May increase workload as parents may raise more concerns in the short term		
	Parents have suggested to staff they would find form useful to document any questions she may have		
	Will increase confidence with medical staff		
	Find it undermining and offensive		
	Should be doing it anyway		
	Doesn’t like idea of parents being able to go straight to PACE		
	Whether it increases safety will depend on the parents		
	Too early to establish whether it will help with communication		

*Implementation:* Out of 51 parents who completed the evaluation questionnaire, 24 parents reported seeing the poster and 20 reported seeing the booklet, however, only three reported actually using the resources. Reasons for non-usage related to lack of awareness or lack of need. Out of 49 staff that completed the evaluation questionnaire, 38 reported being aware of the project and four staff members reported having been involved in parent-initiated discussions using the resources.

*User feedback:* Of the three parents who had used the :Listening To You” resources, two felt that the materials had led to increased confidence in terms of raising concerns and having them listened to. Additional comments from parents suggested that the materials would be of use in the right circumstances (e.g., if they had concerns, were new to the hospital). Of the staff who had seen or used the staff resources, approximately half reported that they were easy to use, gave them confidence to elicit and discuss parental concerns and helped with parent-professional communication. Generally the resources were not perceived as increasing workload. Additional comments from staff were mixed, some saw the tools as having great value, while others perceived them as “undermining and offensive”, suggesting that staff already have the skills required to elicit, discuss and escalate parental concerns. PICU Family Liaison nurses regularly reported to the project team on the effectiveness of the intervention, highlighting in particular, one family who benefitted from the Planning Care Together sheet to restore communication with staff. One nurse commented: “*We used the Planning Care Together form with one long stay family where we identified that there had been a communication breakdown; through using written communication on the forms a couple of times verbal communication was successfully restored*” (Family Liaison Nurse, PICU).

*Incidents and complaints:* Prior to implementation of the “Listening To You” resources, two SIRIs relating to staff not listening to the concerns of parents were recorded. No incidents or complaints had been reported at the end of the pilot. One year on, two incidents had been reported in one clinical area where parents felt that the doctor in charge had not listened to their concerns. However, it was also reported that these incidents had been resolved within the department using the “Listening to You” resources. The positive outcome of these events was that the intervention had been effective in empowering junior staff members to expedite medical review on the basis of parental concern.

*Pediatric Early Warning Scores (parental concerns box):* On the two cardiac wards reviewed, 81% of the parental/nurse concern boxes were completed and of the completed boxes, 4% had documented a parental concern.

## 6. Discussion

Through our “Listening To You” quality improvement project we developed and implemented an innovative approach to listening, incorporating and responding to parental concerns regarding their child’s clinical condition when in hospital. In doing so, channels of communication between professionals and parents/carers have been established which facilitate parents to become active partners in their child’s care. These new processes and resources enable health professionals to elicit and act on the subtle changes that a parent recognises in their child that staff may not witness, despite regular assessments and monitoring equipment. This study contributes to the paucity of literature on family-led approaches to detecting and managing early signs of patient clinical deterioration.

Despite the several examples of positive changes in care that were observed, developing, implementing and evaluating this quality improvement initiative presented a number of challenges. First, limited evidence meant that the intervention development phase took longer than anticipated. At the time, we could not identify any tools or frameworks within the NHS or existing literature, and so the intervention had to be developed from scratch in consultation with service-users. Since completion of the project, a framework for involving parents in the care of a child with a long term condition has been developed through a concept synthesis of Family-Centred Care and partnership-in-care [49]. This model describes three key domains for supporting parental involvement in child healthcare: “(i) valuing parents’ knowledge and experiences; (ii) supporting parents in their role as care giver; and (iii) incorporating parents’ expertise into clinical and psychosocial care” (p. 4). Our development work and resulting intervention offer support for this theoretical framework in an acute care setting. Our intervention also demonstrates the possibility of operationalising concepts of Family-Centred Care in practice, which has previously been presented as challenging [14].

Nevertheless, the impact of having a longer than planned development phase was to limit the length of time devoted to pilot testing and also to force pilot testing during Christmas time; a traditionally busy time in the hospital when routines are disrupted and winter bed pressures are felt. We recognise this as a limitation of our project, particularly as a number of parents and staff reported being unaware of the resources when we conducted the evaluation. However, this finding is not unique to our study. Following the introduction of a Family Activated Rapid Response Team, Ray *et al.* [1], for example, reported that awareness of their new family-led system for initiating medical review ranged from 58% to 6%, depending on the speciality in which families were questioned and when in the month they were asked.

Having limited time to embed the intervention also led to challenges regarding acceptability of our communications bundle to staff. It is already known that involving parents in the care of a child when in hospital is likely to be influenced by the attitudes and actions of health professionals [49]. Professionals have been found to hold unexpressed expectations regarding parental involvement in care [16,50] including that parents should participate, but not to what extent [34]. Concerns regarding increased staff workload, undermining health professional judgement and staff confidence and receptivity to implement change have previously been reported by professionals in relation to the involvement of families in identifying early signs of patient deterioration [2,7]. Similar kinds of barriers were alluded to by health professionals in our stakeholder consultation activities, and then again following intervention implementation, despite positive promotion of parental participation in care throughout the hospital. In areas where staff buy-in was low, Ward Managers reported that examples of positive outcomes from using the resources were helpful for engaging staff in the ethos of the project. Especially valuable were examples describing situations in which staff had struggled to escalate their own or parental concerns to more senior professionals/teams and found the resources helpful for overcoming barriers. Having the backing of the hospital’s Executive Team and identifying a number of ward-based project “champions” further contributed to the acceptability of the intervention. In accordance with research-based recommendations [7], structured training sessions are being planned for staff, along with insertion of the resources into the staff Safety Manual, a handbook distributed by the hospital and carried by all nurses.

Throughout the project, the team identified that the “Listening to You” toolkit needed to be made more accessible to parents in a variety of ways, and that use of the resources needed to be promoted to parents throughout the patient’s hospital stay [5]. Since the pilot phase, therefore, we have continued to inform parents about the resources by incorporating the communications bundle into the hospital’s bedside folders and on the hospital’s website “pre-admission” pages. “Listening To You” posters have also been modified so that they encourage parents to ask staff for a “Planning Care Together” sheet if needed. A number of wards have also incorporated the resources into their existing parent participation activities. For example, Clinical Support Worker’s on the oncology ward have used their parent “tea round” session to offer “Planning Care Together” sheets; Family Liaison nurses on the PICU have incorporated “Listening To You” materials into their weekly “Feedback Friday” parent-professional communication sessions; and the Play Facilitators on each ward have been trained to provide parents with “Listening To You” information and resources. These strategies have helped to promote and embed the intervention, thus ensuring consistency across the hospital and reducing reliance on Ward Managers to disseminate information to parents and staff.

Finally, the evaluation of this pilot intervention was limited in terms of its short pilot phase and small sample size. Part of the premise of this project was to prevent the occasional care deficit which parents had tried to escalate but had not been heard by staff. It is therefore unsurprising that only three parents needed to use the resources during the pilot phase. We recognise that this number would have increased had the pilot phase been longer. However, we also understand that parents do not always feel the need to write down their concerns and that communication may have improved simply by formalising our listening approach. Existing research suggests that families appear to infrequently activate medical response systems and when they do, it is as a result of communication failures rather than critical care deterioration [6]. Nevertheless, implementation and effectiveness of our “Listening to You” resources continues to be monitored as part of the hospital’s standard patient experience and complaints mechanisms.

### Implications for Practice and Future Research

The development of future resources to help parents communicate and escalate concerns that something is wrong with their child when in hospital should be done so in collaboration with families and health professionals. Special attention should be paid to any potential barriers to the acceptability and usability of the intervention. Moreover, resources may need to be developed in a variety of formats with ongoing promotion to maintain awareness throughout the patient’s hospital stay. While our intervention development phase involved input from young people on the wards and at discussions with the hospital’s Young Persons Advisory Group, the tool itself is currently aimed at parents. A future project will be to design a more versatile tool which appeals to both young people (patients) as well as their parents/carers.

Future research is also recommended to conduct more robust and thorough evaluations of such resources, in particular their effect on preventing critical deterioration, parent and staff confidence to raise and escalate concerns and awareness and usability of new initiatives. In addition, consideration should be given to the transferability of any resources across conditions and wards. Although our study did not include an assessment of the resource or cost implications of the intervention, we might anticipate patient benefits from early escalation, but also additional staff resource use. There might also be cost-savings from a reduction in formal complaints; however, the aim of the project was to improve healthcare quality and safety rather than to generate cost-savings. Further research is needed to examine the cost-effectiveness of interventions aiming to elicit and respond to parental concerns regarding child deterioration.

## 7. Conclusions

We developed and implemented an intervention in consultation with parents and staff which aimed to improve communication of parental concerns and appropriate escalation of care when a child is in hospital. The “Listening To You” resources continue to be in use. Further evaluation of how far they empower parents to raise and discuss their concerns with professionals and to ask questions or make comments about their child’s clinical condition in a way that encourages parental participation and facilitates Family-Centred Care and of consequences for the management of patients is required if the resources are to be used more widely.

## Supplementary Materials

The supplementary materials are available online at http://www.mdpi.com/2227-9032/4/1/9/s1.
