# Supplementary material for: Developing a Tool to Support Communication of Parental Concerns When a Child is in Hospital
Source: Healthcare (Basel). 2016 Jan 13;4(1):9. doi: 10.3390/healthcare4010009 (PMC4934543; doi:10.3390/healthcare4010009)
Supplement: Supplementary file 1 [file healthcare-04-00009-s001.zip › healthcare-106628-supplementary-final/healthcare-106628-supplementary table 1 - final.docx]

Supplementary Materials: Developing a Tool to Support Communication of Parental Concerns When a Child is in Hospital

Gemma Heath, Hermione Montgomery, Caron Eyre, Carole Cummins, Helen Pattison and Rachel Shaw

**Table S1.** Characteristics of included studies.

| **Study** | | **Aim** | **Participants** | | | | | | **Data Collection** | **Analysis** |
| --- | --- | --- | --- | --- | --- | --- | --- | --- | --- | --- |
| **First Author  & Date** | **Journal** |  | **Sampling Method** | **No.** | **Adult  Paediatric** | **Nurse  Doctor  Patient** | **Dept. Disease** | **Location** |  |  |
| Ammentorp (2009) [1] | *Scandinavian Journal of Caring Sciences*, 23, 506–517. | To investigate the effect of communication skills training for doctors and nurses on parents’ perceptions of the communication and parents’ satisfaction with the communication. | Not stated | 30 | Adult | Doctors & Nurses & parents of patients | Outpatient | Denmark | Questionnaires | Logistic regressions |
| Arranz (2005) [2] | *Patient Education and Counselling*, *56(2)*,  233–239. | The main purpose of this longitudinal study was to evaluate the effects of this Counseling Training Program on the difficulties that nursing professionals perceived in their work | Opportunistic | Unclear | Adult | Nurses | n/a | Madrid | Training in counselling intervention | Reliability analyses |
| Berlin (2008) [3] | *Scandinavian Journal of Caring Sciences*, *22*, 118–127. | The aim of the study was to theoretically explain the PCHNurses’ core problem regarding their interaction with children and parents of foreign origin. | Theoretical sampling | 15 | Adult | Primary child health nurses | n/a | Sweden | Face-to-face interviews | Informed by grounded theory |
| Boroujeni (2008) [4] | *Journal of Clinical Nursing*, *18*, 2329–2336 | To explore the nurse-patient interaction in terminally ill situations in acute care, focusing on the nurses’ preparation for loss. | Purposive and theoretical sampling | 18 | Adult | Nurses | High risk/Intensive care | Iran | Interviews | Informed by grounded theory |
| Burnard (2004) [5] | *International Journal of Nursing Studies*, *41*. | The aim of the study was to explore the question “in what, if any, ways do Thai cultural issues influence interpersonal communication patterns in Thai nursing and Thai nursing education?” In answering the question, it was hoped that the researchers would be able to illuminate the answers to it through examples from everyday life. | Convenience and purposive sample | 14 | Adult | Nurses | Clinical and nurse educators | Thailand | Observations and interviews | Grounded theory and thematic analysis |

**Table S1.** *Cont.*

| **Study** | | **Aim** | **Participants** | | | | | | **Data Collection** | **Analysis** |
| --- | --- | --- | --- | --- | --- | --- | --- | --- | --- | --- |
| **First Author  & Date** | **Journal** |  | **Sampling Method** | **No.** | **Adult  Paediatric** | **Nurse  Doctor  Patient** | **Dept. Disease** | **Location** |  |  |
| Caris-Verhallen (1997) [6] | *Journal of Advanced Nursing*, *25*, 915–933. | The object is to outline communication research in this field and to highlight the areas in which more research is needed. More specifically, this review seeks to address the following questions: What is the role attributed to communication in various theories of nursing? How do nurses communicate with elderly patients, *i.e.*, what kind of verbal and nonverbal strategies are used in communicating with the elderly? What are the determinants of the quality or quantity of nurse-patient communication? | Review | n/a | Adult | Nurse and Patient interaction | Elderly patients | The Netherlands | Observation | n/a |
| Charlambous (2008) [7] | *European Journal of Oncology Nursing*, *12*, 436–442. | The aim of the study was to investigate the views of patients with cancer and to explore their experiences in relation to quality of nursing care in the Cyprus National Health and Insurance Scheme (private and public). In the light of the patients’ views, the views of the nurses providing services to these patients, and the views of key advocates from the cancer associations were explored. | Purposive sampled | 25 | Adults | Patients | Oncology | Cyprus | Face-to-face interviews & Focus groups | Structural analysis |
|  |  |  |  | 20 | Adults | Nurses | Oncology |  | Face-to-face interviews |  |

**Table S1.** *Cont.*

| **Study** | | **Aim** | **Participants** | | | | | | **Data Collection** | **Analysis** |
| --- | --- | --- | --- | --- | --- | --- | --- | --- | --- | --- |
| **First Author  & Date** | **Journal** |  | **Sampling Method** | **No.** | **Adult  Paediatric** | **Nurse  Doctor  Patient** | **Dept. Disease** | **Location** |  |  |
| Coiera (2002) [8] | *Medical Journal of Australia*, *176*, 415–418. | To measure communication loads on clinical staff in an acute clinical setting, and to describe the pattern of informal and formal communication events. | Opportunistic sampling | 12 | Adult | Nurses & Doctors | Emergency department | New South Wales | Observation | The communication load on clinical staff was measured by the proportion of observed time spent in communication, the proportion of communication events involving concurrent communication asks, and the proportion of interruptions experienced by subjects. The rates of communication events and interruptions and 95% CIs for counted variables. |
| Cornwall (2008) [9] | *European Journal of Oncology Nursing*, *12*, 198–208. | The aim of the study was to evaluate the usefulness of e-mail as a method of communication between patients with lung cancer and their family members, and to identify any advantages and disadvantages of using this method of communication. | Not stated | 2 | Adult | Nurses | Oncology | South East England | Email content, questionnaires and focus group/reflective session | Descriptively and content analysis |
|  |  |  |  | 16 | Adult | Patient & Family members |  |  |  |  |
| DiMatteo (2003) [10] | *Patient Education and Counselling*, *50*, 23–26. | The goal of this paper is to examine emerging issues in consumer-provider communication and patient adherence to cancer prevention, screening, diagnosis, treatment, and coping with survivorship. | Exploratory Review | n/a | n/a | n/a | n/a | n/a | n/a | n/a |

**Table S1.** *Cont.*

| **Study** | | **Aim** | **Participants** | | | | | | **Data Collection** | **Analysis** |
| --- | --- | --- | --- | --- | --- | --- | --- | --- | --- | --- |
| **First Author  & Date** | **Journal** |  | **Sampling Method** | **No.** | **Adult  Paediatric** | **Nurse  Doctor  Patient** | **Dept. Disease** | **Location** |  |  |
| Draper (2002) [11] | *Bioethics*, *16(4)*,  335–352. | We argue that vulnerability does not exclude obligation. We also look at others ways in which patient responsibilities flow from general ethics: for instance, from responsibilities to others and to the self, from duties of citizens, and from the responsibilities of those who solicit advice. Finally, we argue that certain duties of patients counterbalance an otherwise unfair captivity of doctors as helpers. | Exploratory Review | n/a | n/a | n/a | n/a | n/a | n/a | n/a |
| Ferguson (2013) [12] | *Nurse Education in Practice*,  283–287. | How do patients describe their experiences and roles in the interprofessional teams that provide their health care in an acute care medical inpatient-nursing unit? | Not stated | 26 | Adult | Patients (18)  Family members (8) | Inpatient acute medical unit | Canada | Face-to-face interviews | Thematic Analysis |
| Festini (2009) [13] | *Journal of Nursing Scholarship*, *41(2)*,  220–227. | Estimate the frequency of problems perceived by a population of nurses of a major Italian paediatric hospital in delivering care to immigrant children and their families and to explore the perceptions of such nurses regarding nursing care of immigrant children and their families. | Purposive | 129 | Adult | Nurses | Paediatric | Italy | Questionnaires | Descriptive and content analysis  for open  ended questions |
| Forkner-Dunn (2003) [14] | *Journal of Medical Internet Research*, *5(2)*, 8. | Review of the use of the Internet by physicians. | Exploratory Review | n/a | n/a | n/a | n/a | n/a | n/a | n/a |
| Hafsteindottir (1996) [15] | *Intensive and critical care nursing*, *12*, 261–271. | To describe the patients’ experiences of communication during the RT period. The research question was: How does the respirator treated patient experience communication? | Not stated | 8 | Adult | Patients | Intensive  care unit | Iceland | Face-to-face interviews | Phenomenological |

**Table S1.** *Cont.*

| **Study** | | **Aim** | **Participants** | | | | | | **Data Collection** | **Analysis** |
| --- | --- | --- | --- | --- | --- | --- | --- | --- | --- | --- |
| **First Author  & Date** | **Journal** |  | **Sampling Method** | **No.** | **Adult  Paediatric** | **Nurse  Doctor  Patient** | **Dept. Disease** | **Location** |  |  |
| Han (2005) [16] | *Psycho-Oncology*, *14*, 318–330. | Examined psychosocial factors pertaining to being proactive during medical encounters that we hypothesized could be associated with difficulty interacting and communicating with physicians and nurses on the part of breast cancer patients, and also with these women’s overall satisfaction with their physicians. | Not stated | 352 | Adult | Patients | Oncology  Breast cancer | United States | Questionnaires  The CARES: Medical Interaction Subscale  The Impact of Illness on your life scale  The Stanford Emotional self-efficacy scale  The Impact of event scale  The social network and support assessment | Cross-sectional analysis |
| Kai (1996) [17] | *British Medical Journal*, *313*, 983–986 | Identify what worries parents when their children become acutely ill and to understand what motivates their concerns. | Purposive | 95 | Adult | Parents of acutely ill children | Paediatrics | UK | Face-to-face interviews  Focus groups | Informed by grounded theory |
| Kruijver (2000) [18] | *Patient Education and Counselling*, *39(1)*,  129–145. | What are the characteristics of the communication training programs evaluated for nurses with regard to the following independent variables: participants’ background characteristics, professional background of the teachers, characteristics of the training program? What research methods are used to evaluate the outcomes of the training? What are the process-oriented and effect-oriented outcomes of the training programs? | Systematic review | n/a | n/a | n/a | n/a | n/a | n/a | n/a |
| Langewitz (2002) [19] | *British Medical Journal*, *325*, 682–683. | Examined how long it would take outpatients at a tertiary referral centre to indicate that they have completed their story, *i.e.*, with a statement such as “that’s all doctor” if uninterrupted by their doctors. | Opportunistic | 330 | Adult | Patients & Physicians | University hospital | Germany | Stopwatch | Descriptive |

**Table S1.** *Cont.*

| **Study** | | **Aim** | **Participants** | | | | | | **Data Collection** | **Analysis** |
| --- | --- | --- | --- | --- | --- | --- | --- | --- | --- | --- |
| **First Author  & Date** | **Journal** |  | **Sampling Method** | **No.** | **Adult  Paediatric** | **Nurse  Doctor  Patient** | **Dept. Disease** | **Location** |  |  |
| Liu (2005) [20] | *Journal of Advanced Nursing*, *52(3)*,  262–270. | This paper reports the findings of a study exploring the experiences and expectations of patients with cancer of supportive communication in the context of Chinese culture. | Convenience | 20 | Adult | Patients | Oncology | Beijing, China | Semi-structured interviews | Content analysis |
| Mann (2012) [21] | *ANZ Journal of Surgery*, *82*, 671–674. | The aim of the current study was to retrospectively audit all complaints made against the General Surgical Department of a large university hospital over a 12-month period. In particular, the study aimed to identify the frequency of complaints, the number that give rise to medico-legal claims and the number that relate to patient safety events | Prospective | 113 | Adult | Patients | University hospital | Leicester, England | Complaint letters, log of telephone conversations and minutes of meetings. | Descriptive |
| Norgaard (2012) [22] | *Scandinavian Journal of Caring Sciences*, *26*, 698–704. | To investigate whether adult orthopaedic patients’ evaluation of the quality of care had improved after a communication skills training course for healthcare professionals. | Not stated | 3133 | Adult | Patients & nurses, doctors and medical secretaries | Orthopaedic surgery ward | Denmark | Questionnaire pre and post training course on communication skills for health care professionals | Cross-sectional analysis |
| Ong (2000) [23] | *Patient Education and Counseling*, *41*, 145–156. | The following re-search questions will be addressed:  1. What is the relationship between doctors’ and patients’ communication during the initial oncological consultation and patients’ quality of life and satisfaction?  2. What is the relationship between oncologists’ patient-centredness and patients’ quality of life and satisfaction? | Not stated | 96 | Adult | Patient | Oncology | Amsterdam | Questionnaires  RIAS  Quality of life  Patients satisfaction with the consultation | Cross-sectional analysis and  Content Analysis |
| Rhodes (2004) [24] | *Annals of Emergency Medicine*, *44(3)*,  262–267. | This small pilot study attempts to characterize communication in an urban academic medical center ED with regard to the timing and nature of the history and physical examination and discharge instructions. | Convenience sample | 93 nonemergency patients | Adult | Patients | Emergency Department | USA | Audiotaped consultations with nurses and physicians | Descriptive methods |

**Table S1.** *Cont.*

| **Study** | | **Aim** | **Participants** | | | | | | **Data Collection** | **Analysis** |
| --- | --- | --- | --- | --- | --- | --- | --- | --- | --- | --- |
| **First Author  & Date** | **Journal** |  | **Sampling Method** | **No.** | **Adult  Paediatric** | **Nurse  Doctor  Patient** | **Dept. Disease** | **Location** |  |  |
| Richardson (2006) [25] | *European Journal of Oncology Nursing*, *10*, 93–101. | This study arose from a concern to explore more fully the views of professionals currently working in health and social care and to consider their needs for training. | Convenience sample | 28 | Adult | Nurses (22)  Consultant Physician (1)  Social Workers (2)  Occupational Therapist (1)  Welfare rights officer (1)  Information worker (1) | Oncology/palliative | UK | Focus groups | Thematic analysis |
| Siyambalapitiya (2006) [26] | *International Journal of Nursing Practice*, *13*, 107–110. | Audit patient complaints made about medical care in a National Health Service District general hospital over a 22-month period. | Prospective | 183 complaints | Adult | Patients | Inpatient admissions | UK | Auditing complaints | Unclear |
| Straka (2010) [27] | *Journal of Pediatric Nursing*, *25,* 33–34. | To trial a wireless telephones were implemented on an adolescent medical unit for a 6-week trial period | Convenience | 27 | Adult | Patient’s family & nurses | Paediatric acute care inpatient unit | USA | Patient survey | Descriptive |
| Tay (2011) [28] | *International Journal of Evidence-Based Healthcare*, *9*, 131–164. | To establish the best available evidence regarding the factors affecting effective communication between Registered nurses and inpatient cancer adults. | Systematic review | n/a | n/a | n/a | n/a | n/a | n/a | n/a |

**Table S1.** *Cont.*

| **Study** | | **Aim** | **Participants** | | | | | | **Data Collection** | **Analysis** |
| --- | --- | --- | --- | --- | --- | --- | --- | --- | --- | --- |
| **First Author  & Date** | **Journal** |  | **Sampling Method** | **No.** | **Adult  Paediatric** | **Nurse  Doctor  Patient** | **Dept. Disease** | **Location** |  |  |
| Turner (2009) [29] | *European Journal of Cancer*, *45*, 1798–1806. | This study aimed to enhance the capacity of oncology nurses to provide supportive care for patients with advanced cancer who have dependent children. | Not stated | 35 | Adult | Nurses | Oncology | Australia | Questionnaires  Measures of burnout and psychological morbidity  Measures of perceived stress, confidence and attitudes  Assessment of knowledge—clinical vignettes Assessment of skills—simulated patient interviews | Descriptive analyses and Wilcoxon paired ranks tests |
| Woloshynowych (2007) [30] | *The Practice of Emergency Medicine/Original Research*, *50(4)*,  407–413. | The primary aims of the present study were to use the communication observation method to investigate the communication load of the nurse in charge of the ED and to build on this method by collecting additional information that would help us to interpret the data, such as patient throughput and staffing levels | Convenience sample | 11 | Adult | Nurses | Emergency Department | London | Observational study | Descriptive analyses |

**Reference**

1. Ammentorp, J.; Sabroe, S.; Kofoed, P.E.; Mainz, J. Effects of a communication course for clinicians on parents’ perception of care- a randomized controlled trial. *Scand. J. Caring Sci.* **2009**, *23*, 506–517.
2. Arranz, P.; Ulla, S.M.; Ramos, J.L.; Rincon, C.D.; Lopez-Fando, T. Evaluation of a counselling training program for nursing staff. *Patient Edu.* *Couns.* **2005**, *56*, 233–239.
3. Berlin, A.; Hylander, I.; Tornkvist, L. Primary child health care nurses’ assessment of health risks in children of foreign origin and their parents—A theoretical model. *Scand. J. Caring Sci.* **2008**, *22*, 118–127.
4. Boroujeni, A.Z.; Mohammadi, R.; Oskouie, S.F.H.; Sandberg, J. Iranian nurses’ preparation for loss: Finding a balance in end-of-life care. *J**. Clin. Nurs.* **2008**, *18*, 2329–2336.
5. Burnard, P.; Naiyapatana, W. Culture and communication in Thai nursing: A report of an ethnographic study. *Int. J. Nurs. Stud.* **2004**, *41*, 755–765.
6. Caris-Verhallen, W.M.C.M.; Kerkstra, A.; Bensing, J.M. The role of communication in nursing care for elderly people: A review of the literature. *J. Adv. Nurs.* **1997**, *25*, 915–933.
7. Charalambous, A.; Papadopoulos, R.; Beadsmoore, A. Listening to the voices of patients with cancer, their advocates and their nurses: A hermeneutic-phenomenological study of quality nursing care. *Eur. J. Oncol. Nurs.* **2008**, *12*, 436–442.
8. Coiera, E.W.; Jayasuriya, R.A.; Hardy, J.; Bannan, A.; Thorpe, M.E.C. Communication loads on clinical staff in the emergency department. *Med. J. Aust.* **2002**, *176*, 415–418.
9. Cornwall, A.; Moore, S.; Plant, H. Embracing technology: patients’, family members’ and nurse specialists’ experience of communicating using e-mail. *Eur. J. Oncol. Nurs.* **2008**, *12*, 198–208.
10. DiMatteo, M.R. Future directions in research on consumer-provider communication and adherence to cancer prevention and treatment. *Patient Edu. Couns.* **2003**, *50*, 23–26.
11. Draper, H.; Sorell, T. Patients’ responsibilities in medical ethics. *Bioethics* **2002**, *16*, 335–352.
12. Ferguson, L.M.; Ward, H.; Card, S.; Sheppard, S.; McMurtry, M. Putting the “patient” back into patient-centred care: An education perspective. *Nurse Edu. Pract.* **2013**, *13*, 283–287.
13. Festini, F.; Focardi, S.; Bisogni, S.; Mannini, C.; Neri, S. Providing transcultural to children and parents: an exploratory study from Italy. *J. Nurs. Scholarsh.* **2009**, *41*, 220–227.
14. Forkner-Dunn, J. Internet-based patient self-care: The next generation of health care delivery. *J. Med.* *Internet Res.* **2003**, doi:10.2196/jmir.5.2.e8.
15. Hafsteindottir, T.B. Patient’s experiences of communication during the respirator treatment period. *Intensive Crit. Care Nurs.* **1996**, *12*, 261–271.
16. Han, T.W.; Collie, K.; Koopman, C.; Azarow, J.; Classen, C.; Morrow, G.R.; Michel, B.; Brennan-O’Neil, E.; Spiegel, D. Breast cancer and problems with medical interactions: Relationships with traumatic stress, emotional self-efficacy, and social support. *Psycho-Oncology* **2005**, *14*, 318–330.
17. Kai, J. What worries parents when their preschool children are acutely ill, and why: A qualitative study. *Br. Med. J.* **1996**, *313*, 983–986.
18. Kruijver, I.P.M.; Kerkstra, A.; Francke, A.L.; Bensing, J.M.; van de Wiel, H.B.M. Evaluation of communication training programs in nursing care: a review of the literature. *Patient Edu. Couns.* **2000**, *39*, 129–145.
19. Langewitz, W.; Denz, M.; Keller, A.; Kiss, A.; Ruttimann, S.; Wossmer, B. Spontaneous talking time at start of consultation in outpatient clinic: Cohort study. *Br. Med. J.* **2000**, *325*, 682–683.
20. Liu, J.E.; Mok, E.; Wong, T. Perceptions of supportive communication in Chinese patients with cancer: Experiences and expectations. *J. Adv. Nurs.* **2005**, *52*, 262–270.
21. Mann, C.D.; Howes, J.A.; Buchana, A.; Bowrey, D.J. One-year audit of complaints made against a university hospital surgical department. *ANZ J. Surg.* **2012**, *82*, 671–674.
22. Norgaard, B.; Kofoed, P.E.; Kyvik, K.O.; Ammentorp, J. Communication skills training for health care professionals improves the adult orthopaedic patient’s experiences of quality of care. *Scand. J. Caring Sci.* **2012**, *26*, 698–704.
23. Ong, L.M.L.; Visser, M.R.M.; Lammes, F.B.; de Haes, J.C.J.M. Doctor-patient communication and caner patients’ quality of life and satisfaction. *Patient Edu. Couns.* **2000**, *41*, 145–156.
24. Rhodes, K.V.; Vieth, T.; He, T.; Miller, A.; Howes, D.S.; Bailey, O.; Walter, J.; Frankel, R.; Levinson, W. Resuscitating the physician-patient relationship: emergency department communication in an academic medical center. *Ann. Emerg. Med.* **2004**, *44*, 262–267.
25. Richardson, A.; Thomas, V.N.; Richardson, A. “Reduced to nots and smiles”. Experiences of professionals caring for people with cancer from black and ethnic minority groups. *Eur. J. Oncol. Nurs.* **2006**, *10*, 93–101.
26. Siyambalapitiya, S.; Caunt, J.; Harrison, N.; White, L.; Weremczuk, D.; Fernando, D.J.S. A 22 month study of patient complaints at a national health service hospital*. Int. J. Nurs. Pract.* **2006**, *13*, 107–110.
27. Straka, K.L. Wireless telephone systems and the impact on patient satisfaction scoring: A pilot study. *J.* *Pediatr. Nurs.* **2010**, *25*, 33–34.
28. Tay, L.H.; Hegney, D.; DNurs, E.A. Factors affecting effective communication between registered nurses and adult cancer patients in an inpatient setting: A systematic review. *Int. J. Evid.* *Based Healthcare* **2011**, *9*, 131–164.
29. Turner, J.; Clavarino, A.; Butow, P.; Yates, P.; Hargraves, M.; Connors, V.; Hausmann, S. Enhancing the capacity of oncology nurses to provide supportive care for parents with advanced cancer: Evaluation of an educational intervention. *Eur. J. Cancer* **2009**, *45*, 1798–1806.
30. Woloshynowych, M.; Davis, R.; Brown, R.; Vincent, C. Communication patterns in a uk emergency department. *Pract. Emerg. Med.* *Orig. Res.* **2007**, *50*, 407–413.

© 2016 by the authors; licensee MDPI, Basel, Switzerland. This article is an open access article distributed under the terms and conditions of the Creative Commons by Attribution (CC-BY) license (http://creativecommons.org/licenses/by/4.0/).
