# Supplementary material for: Developing a Tool to Support Communication of Parental Concerns When a Child is in Hospital
Source: Healthcare (Basel). 2016 Jan 13;4(1):9. doi: 10.3390/healthcare4010009 (PMC4934543; doi:10.3390/healthcare4010009)
Supplement: Supplementary file 1 [file healthcare-04-00009-s001.zip › healthcare-106628-supplementary-final/Listening to you parent guide.pdf]

Talk to us – we are listening

- There is no such thing as a silly question.
- Asking questions will NOT affect your child's care.
- Don't worry if you get upset, it's ok.
- If you're not sure what the nurses or doctors are doing, ask them to explain it to you.
- If you think something is wrong, don't wait to talk to us.

Are you worried about your child? Talk to your nurse

- Does your child seem different to their normal self?
- Is it something you can see? Is it something you feel?
- Is it something your child is doing/not doing?

Thinking about the things above might help you describe your worry to staff. The diagram overleaf may help you too. Use the "Talking to staff about your worries: Planning care together" form to communicate your worries to staff.

"You might spot something sooner than the health professionals"

Most of the time when your child is in hospital you won't feel the need to alert someone about your child's condition. Sometimes you may be worried and this leaflet will help you to talk to staff.

No one knows your child better than you. You have an important role to play in the care of your child and we will listen to you.

"I don't know what it is but my child is 'just not right'"

Our Birmingham Children's Hospital Trust Values reflect how we will listen to you

You can **Trust** us to listen to your worries and feelings.

We are **Commited** to listening to you and involve you in decisions relating to your child.

We **Respect** the knowledge you have about your child and will listen to you.

We demonstrate **Compassion** by listening and understanding worries you have about your child.

We support you to have **Courage** to respectfully challenge us and we have courage to listen to you.

We have heard you

Birmingham Children's Hospital NHS
NHS Foundation Trust

Listening to you

A guide to help parents discuss worries or concerns about their child with Health Professionals.

You know your child best and we will listen to you.

The Health Foundation
Inspiring Improvement

We Will LISTEN To You

Look at your child

Use this diagram as a guide to help you describe what is making you worried.

Is your child:
- Confused or muddled
- Irritable
- In an odd mood
- Using a different cry
- More sleepy
- Quieter than normal
- Saying that they do not feel right?
- Behaving differently

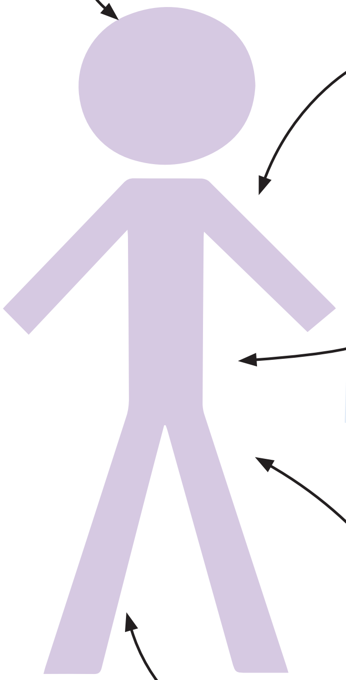

Are there changes to your child's breathing?
- Faster
- Slower
- Working harder
- Making noises

How does your child look?
- More pale than usual
- More flushed than usual
- Blotchy
- Rashes/marks on body
- Puffy
- Sweaty

Are there differences in your child's:
- Wee /poo
- Eating
- Drinking
- Vomiting

Is your child:
- Moving around a lot or being very still
- Appears to be in pain or discomfort
- Saying somewhere hurts

These are just examples; if you see any other signs please tell us what they are, talk to your nurse or doctor.

What to do next, you may not need to progress any further than step 1.

Step 1 – **Talk to your nurse**

We will:
Assess your child
Do observations
Record on PEWS chart

We will:
Escalate your concern

We will:
Keep you informed and involved in any decisions

Step 2 – **Talk to the nurse in charge**

Step 3 – **Talk to a doctor on a ward round or ask a nurse to call a doctor**

Definitions:

PEWS = Paediatric Early Warning Signs - the coloured chart that the nurses write the observations on

Escalate your concern - raise your worry to get your child re-assessed by a senior member of staff.

You may use these tips to help you describe your worry to staff

STARTING THE CONVERSATION

Your nurse or doctor may be attending to another patient so make sure that you have the full attention of the nurse / doctor before discussing your worries.

"I need to talk to you about my child as I am worried"

MY CONCERN

Tell the nurse / doctor that you feel that something is not right and what your worry is. Try and give them as much information as possible. You may find your notes or stick diagram helpful.

"I am worried that he is sleeping too much"

REASON / SOLUTION

Tell them if you have an idea of what is going wrong and what will help

"He has been sleepy since his pain medication was changed"

AGREEMENT

The nurse / doctor will discuss the plan of action with you and this will be recorded in the notes

"The nurse has come back to me to say they have spoken to the doctor who will review his pain medicine and get back to us"
