# Supplementary material for: Developing a Tool to Support Communication of Parental Concerns When a Child is in Hospital
Source: Healthcare (Basel). 2016 Jan 13;4(1):9. doi: 10.3390/healthcare4010009 (PMC4934543; doi:10.3390/healthcare4010009)
Supplement: Supplementary file 1 [file healthcare-04-00009-s001.zip › healthcare-106628-supplementary-final/Listening to you poster.pdf]

Birmingham Children's Hospital NHS
NHS Foundation Trust

Listening to you

A guide to help parents discuss worries or concerns about their child with Health Professionals. You may not need to progress any further than step 1.

Step 1

Talk to your nurse

We will: Assess your child
Do observations
Record on PEWS chart

We will: Escalate your concern

We will: Keep you informed and involved in any decisions

Step 2

Talk to the nurse in charge

Step 3

Talk to a doctor on a ward round or ask a nurse to call a doctor

The Health Foundation
Inspiring Improvement

We Will LISTEN To You
