# Supplementary material for: Developing a Tool to Support Communication of Parental Concerns When a Child is in Hospital
Source: Healthcare (Basel). 2016 Jan 13;4(1):9. doi: 10.3390/healthcare4010009 (PMC4934543; doi:10.3390/healthcare4010009)
Supplement: Supplementary file 1 [file healthcare-04-00009-s001.zip › healthcare-106628-supplementary-final/Listening to you staff.pdf]

How parents' concerns about their child are managed:

On admission, during PEWS observations, at the beginning/end of your shift and during other contact with patients you will ask if parents have any concerns. At other times they may raise concerns directly with you, which you may or may not already be aware of. This flowchart will guide you to manage and escalate concerns.

Step 1

Listen to parents

↓

Check understanding of what the parents concern is

↓

Feedback to parents on obs

↓

Let parents know you have escalated their concern

↓

Involve parents in decisions and agree on what will be done

↓

Step 2

Assess
Check Obs
Record on PEWS

↓

Step 3

Escalate concern to Dr / Nurse in charge

↓

Step 4

PACE

There may be occasions when parents will escalate concerns directly to other colleagues. If this happens please support and respect their decision.
You will be supported by the Trust to escalate your concerns to the PACE Team

Our Birmingham Children's Hospital Trust Values reflect how we will listen to you

You can **Trust** us to listen to your worries and feelings.

We are **Commited** to listening to you and involve you in decisions relating to your child.

We **Respect** the knowledge you have about your child and will listen to you.

We demonstrate **Compassion** by listening and understanding worries you have about your child.

We support you to have **Courage** to respectfully challenge us and we have courage to listen to you.

Birmingham Children's Hospital NHS
NHS Foundation Trust

Staff

What is my role in "Listening to you"

My role is to hear what parents have to say

The Health Foundation
Inspiring Improvement

We Will LISTEN To You

Useful tools for staff for listening and talking to parents:

Remember the importance of introduction and the skills for developing rapport. This will provide opportunities for parents to talk to you.

SUN

S Stop & listen
U Understanding - check your understanding of parent's concern
N Narrate – describe what you're doing & why

Active listening

Clarify
Eg. "Have I got that right?"

Summarise
Summarise what you have heard and what actions you are going to take
Work in partnership to come up with a plan of action

Reflect back
Use parents own words to reflect back what you have heard

Listen for feelings
Watch their non-verbal behaviour

Probe for information
Ask for the specifics. Eg "When did this happen?" or "How many times?"

Show interest
Give parents your undivided attention to show that you are listening to them
Ask them what they would like to happen, if they have any ideas

Signal encouragement
Use non-verbal behaviours such as nodding

Decide if this is an emergency and if so, take appropriate action

Some additional points for listening and responding to parents:

- Feedback to parents, even if it is to tell them there is a delay to show that you have listened to them and are doing something
- Don't take their anger personally. They own their own emotions. However, try to establish what is triggering their higher levels of anxiety
- Consider language barriers – use the correct interpreting service
- Remember to ensure that the language you use reflects the understanding of the person you are talking to - when parents are using medical terminology check that they understand what it means
- Remember that if you involve parents they will feel engaged and empowered and will work better with you
- Be non-judgemental
- Consider each concern individually
- Avoid medical jargon

Use SBAR to help elicit information from parents and to escalate to doctors

Situation
What's happening now? What is the parent's worry?

Background
How long had it been like this?

Assessment
Check the child using the ABCD assessment
PEWS observations

Recommendation
What is your recommendation?
Describe to parents what you are going to do and what they can expect to happen

IPASS – use this to escalate the child's care to a doctor/PACE

Illness severity – what is your assessment of illness severity from your own & parents' concern?

Patient summary – what is the patient's current state?

Action list – what actions have you taken/need to be taken?

Situation awareness & contingency planning – other contributing factors/what will you do if this doesn't work?

Synthesis by receiver – understanding of those receiving the information.
