# Supplementary material for: Developing a Tool to Support Communication of Parental Concerns When a Child is in Hospital
Source: Healthcare (Basel). 2016 Jan 13;4(1):9. doi: 10.3390/healthcare4010009 (PMC4934543; doi:10.3390/healthcare4010009)
Supplement: Supplementary file 1 [file healthcare-04-00009-s001.zip › healthcare-106628-supplementary-final/Planning care together.pdf]

Talking to staff about your worries:
Planning care together

Birmingham Children's Hospital NHS
NHS Foundation Trust

IF YOU FEEL THIS IS AN EMERGENCY PLEASE CALL FOR IMMEDIATE HELP FROM ANY MEMBER OF STAFF

My child's name is:

Today's date:

What I would like you to know:
Sharing my worries, concerns or observations...

What I would like us to do next:
Planning the journey together...

You can share this information with staff if you wish. This information has been shared with the:

☐ Bedside Nurse: Name .... Signed .... Date ___ / ___ / ___

☐ Medical Team: Name .... Signed .... Date ___ / ___ / ___

Please file with the PEWS charts. To be used in conjunction with the **"Listening to You"** project
