# Supplementary material for: Developing a Tool to Support Communication of Parental Concerns When a Child is in Hospital
Source: Healthcare (Basel). 2016 Jan 13;4(1):9. doi: 10.3390/healthcare4010009 (PMC4934543; doi:10.3390/healthcare4010009)
Supplement: Supplementary file 1 [file healthcare-04-00009-s001.zip › healthcare-106628-supplementary-final/Systematic Review - PRISMA flowchart.docx]

**PRISMA Flowchart**
